# Prospective Molecular Targets for Natural Killer Cell Immunotherapy against Glioblastoma Multiforme

**DOI:** 10.3390/cells13181567

**Published:** 2024-09-17

**Authors:** Luke C. Cooksey, Derek C. Friesen, Enrique D. Mangan, Porunelloor A. Mathew

**Affiliations:** 1 Texas College of Osteopathic Medicine, University of North Texas Health Science Center, Fort Worth, TX 76107, USA; lukecooksey@my.unthsc.edu (L.C.C.); derekfriesen@my.unthsc.edu (D.C.F.); enriquemangan@my.unthsc.edu (E.D.M.); 2 Department of Microbiology, Immunology and Genetics, School of Biomedical Sciences, University of North Texas Health Science Center, Fort Worth, TX 76107, USA

**Keywords:** immunotherapy, natural killer (NK) cells, glioblastoma, immune checkpoints

## Abstract

Glioblastoma multiforme (GBM) is the most common type of primary malignant brain tumor and has a dismal overall survival rate. To date, no GBM therapy has yielded successful results in survival for patients beyond baseline surgical resection, radiation, and chemotherapy. Immunotherapy has taken the oncology world by storm in recent years and there has been movement from researchers to implement the immunotherapy revolution into GBM treatment. Natural killer (NK) cell-based immunotherapies are a rising candidate to treat GBM from multiple therapeutic vantage points: monoclonal antibody therapy targeting tumor-associated antigens (TAAs), immune checkpoint inhibitors, CAR-NK cell therapy, Bi-specific killer cell engagers (BiKEs), and more. NK therapies often focus on tumor antigens for targeting. Here, we reviewed some common targets analyzed in the fight for GBM immunotherapy relevant to NK cells: EGFR, HER2, CD155, and IL-13Rα2. We further propose investigating the Lectin-like Transcript 1 (LLT1) and cell surface proliferating cell nuclear antigen (csPCNA) as targets for NK cell-based immunotherapy.

## 1. Introduction

Malignant gliomas are the most frequently occurring type of primary brain cancer in adults. A subset of malignant gliomas, termed glioblastoma and classified according by the World Health Organization (WHO) as Isocitrate Dehydrogenase (IDH)-wild-type glioblastoma in the most recently updated guidelines of 2021, is the most common adult-type diffuse glioma [1]. It arises in the cerebral hemispheres and frequently involves the corpus callosum. The tumor is characterized by rapid and diffuse growth, microvascular proliferation, central areas of necrosis, and histological heterogeneity. Despite the development of some novel therapies, GBM remains an overall deadly disease with a median survival of approximately 12 to 23 months from diagnosis [1]. It has a median age of onset of 65 years [2]. GBM often exhibits five genetic mutations that are viewed as molecular markers for identification and prognosis: (1) IDH mutation status; (2) O-6-methylguanine-DNA methyltransferase (MGMT) promoter methylation status; (3) Epidermal Growth Factor Receptor (EGFR) amplification; (4) chromosome 7 gain and chromosome 10 loss; and (5) Telomerase Reverse Transcriptase (TERT) promoter mutations [1,3]. MGMT is an enzyme involved in DNA repair and its methylation status significantly influences outcomes and treatment plans. Unmethylated MGMT exhibits a worse prognosis compared to methylated MGMT. Temozolomide (TMZ) chemotherapy given to patients with methylated GBM status significantly increases overall survival as compared to patients with unmethylated MGMT status. Patients with unmethylated GBM status typically do not respond to TMZ therapy [4]. The current standards for GBM treatment align with the three pillars of oncology therapy: maximal safe surgical resection of the tumor followed by concurrent radiation therapy and TMZ chemotherapy [5]. While some studies have investigated the use of lomustine, bevacizumab, and combinations of other therapies to conflicting results, more research is still needed to promote the use of these therapies as standard for GBM [1,6,7,8]. To improve the overall survival of patients with GBM, many researchers are attempting to bring the immunotherapy revolution to be a part of GBM therapy. However, even after much effort, the incorporation of immunotherapy into GBM treatment has yielded disappointing results [9]. Many scientists believe the challenges that GBM immunotherapy has encountered stem from some of the innate complexities intrinsic to and surrounding the tumors. These issues include the blood–brain barrier, the highly immunosuppressive tumor microenvironment, the immune “privileged” status of the central nervous system, and the molecular heterogeneity of GBM tumors [10]. However, instead of giving up on the use of immunotherapy to treat GBM, we propose to expand the investigations of GBM immunotherapy and to increase creativity in designing novel therapies. Here, we focus on using natural killer cells to target and destroy GBM cells through various therapeutic means that utilize the innate anticancer activities of natural killer cells.

## 2. Natural Killer Cells

Natural killer (NK) cells arise from hematopoietic stem cells (HSCs) through the lymphocyte cell lineage. NK cells play a crucial role in the innate immune system, participating in the identification of cells that have been infected by viruses or have become cancerous [11,12]. Unlike T cells, NK cells do not require prior sensitization to specific antigens or the presence of major histocompatibility complex (MHC) molecules for activation. NK cell activation is regulated by the balance of inhibitory and activating signals received from various receptors [13,14,15]. While inhibitory receptors recognize self-MHC molecules, activating receptors engage stress-induced ligands on target cells [16,17,18]. This unique ability allows NK cells to identify and eliminate infected or transformed cells without the need for prior exposure, MHC matching, or co-stimulatory signals [11]. NK cells act as crucial intermediaries between innate and adaptive immunity, responding to the cytokine microenvironment and Type I interferon [19,20]. Triggered by Type I IFN and the cytokine milieu, NK cells unleash potent effector functions, influencing immune responses [21,22,23]. They engage in intricate crosstalk with dendritic cells [24]. Furthermore, in lymph nodes, NK cells contribute to T helper 1 (TH1) cell priming through IFN-γ induction [12,25,26,27,28]. Notably, NK cells demonstrate some memory-like properties, participating in IFN-mediated recall responses, underscoring their pivotal role in orchestrating immune reactions to viruses and cancer [29]. The precursors and transitional stages responsible for generating NK cells are characterized by distinct surface markers that correspond to specific lineages. The characteristics and attributes known to define NK cells are governed by the controlled expression of essential transcription factors during development: the T-box transcription factors T-bet and Eomesodermin (EOMES) [11,30,31,32,33,34]. In humans, natural killer cells constitute approximately 5–20% of the total circulating lymphocyte population [11]. Among lymphocytes, NK cell percentages vary from roughly 2–5% in the spleens and bone marrows of inbred laboratory mice up to twice that in mice captured from the wild (with distinctive roles and surface antigens) [11]. Human NK cells are often characterized by CD56 (NCAM) surface expression and, under activated conditions, the stimulating Fc receptor CD16 [11,35,36,37]. From this rather basic state, two distinct subsets are identified based on these two markers within the CD56+ NK cell population: CD56^bright^ and CD56^dim^ cells [11,37,38]. CD56^bright^ NK cells are typically found in secondary lymphoid tissues and are responsible for the potent production of cytokines in response to tumor cell proliferation or viral infection, making them an important defense against new cancers [39]. CD56^dim^ cells predominantly circulate in peripheral blood and carry out cytotoxic functions by releasing IFN-γ, perforin, and granzyme [40].

### 2.1. NK Cell Activity

#### 2.1.1. NK Cell Inhibition

The predominant mechanism by which NK cells are known to be inhibited is via interactions with host MHC class I molecules. This inhibition is mediated through activities of various NK surface receptors with MHC class I molecules, especially killer cell inhibitory receptors (KIRs) [41]. Many of the inhibitory receptors for NK cells are associated with the MHC class I receptor, but there have been many independent inhibition signals discovered in the past few decades [13,42]. The inhibitory receptors consist of an immunoreceptor tyrosine-based inhibitory motif (ITIM), which is characterized by the sequence (I/L/V/S)XYXX(L/V) (with X representing any amino acid, and slashes indicating alternative amino acids at a given position) [13,43]. Upon interaction between ITIM-containing receptors and their ligands, the tyrosine residue undergoes phosphorylation, most likely by an Src family kinase [13,44]. This phosphorylation event leads to the recruitment of the lipid phosphatase SHIP-1, which breaks down phosphatidylinositol-3,4,5-trisphosphate into phosphatidylinositol-3,4-bisphosphate or the tyrosine phosphatases SHP-1 or SHP-2 [13,42,45]. Inhibitory NK receptors enlist the previously mentioned phosphatases to the points of contact between NK cells and targets [13]. These tyrosine phosphatases block NK cell responses by removing phosphates from the tyrosine kinases associated with stimulatory NK receptors [13]. These phosphatases effectively terminate processes that lead to the effector functions of the NK cells [13].

#### 2.1.2. NK Cell Activation

NK cells do not possess a dominant antigen receptor for their cytotoxic activation; unlike T and B cells which possess a single receptor to govern most of their activation and development [13,46,47]. NK cells require the sum of activating signals to exceed the influence of inhibitory receptors to have an effective response [13]. Past experiments, such as those from Bryceson and Long, have shown that agonist antibodies binding a single receptor type, other than CD16, are unable to elicit cytokine secretion or cytolytic activity from the NK cell [11,13,17,48,49]. Activation of the NK cell appears to require simultaneous cross-linking of different receptors, often with additive or even synergistic effects [48]. Major cytotoxic activation of the NK cells requires a synergistic interaction of activating pathways, including three major biochemical pathways of activating receptors identified by Lanier: CD244 receptor systems, ITAM receptor complexes, and DAP10-associated to NKG2D receptor complexes [13,16,48].

### 2.2. Targeting NK Activity against Cancer

NK cells are effective recognizers of tumor cells lacking MHC class I on their surface, yet mostly spare tumor cells that do express MHC class I [13,50,51]. This is an important defense against cancer since CD8+ cytotoxic T cells are unable to successfully recognize most tumor cells that do not display MHC class I [52,53]. When exposed to tumor cells, NK cells undertake two principal functions: triggering degranulation to induce cytolytic apoptosis in unidentified cells and releasing cytokines to modulate the overall immune response [11,37]. The cytokines discharged by NK cells mainly encompass IFN-γ, TNF-α, and Granulocyte–Macrophage Colony-Stimulating Factor (GM-CSF) [11,54]. These cytokines, in conjunction with IL-4, IL-7, and IL-12, prompt the recruitment and heightened activity of hematopoietic cells, leading to an amplified immune response [11,49].

### 2.3. NK Cells and Glioblastoma

Activated natural killer cells access the central nervous system (CNS) by crossing the blood–brain barrier (BBB) and entering the cerebrospinal fluid (CSF) via the choroid plexus [55,56,57]. Their entry into the CNS is mainly composed of the immature NK cell subtype CD56^bright^ human NK cells [58]. Once in the CNS parenchyma, NK cells are important in providing immunosurveillance and controlled cytotoxic functions [58]. In the context of CNS disorders, particularly brain tumors, NK cells contribute to cancer immunosurveillance by targeting cancer cells, including highly malignant stem cells [58,59,60]. They play a role in eliminating senescent cells, providing defense against viral infections, and regulating immune response in the CNS [58,61]. Studies indicate that activated CD16-positive NK cells are associated with improved survival in GBM patients [61,62]. The presence of these activated NK cells correlates with lower-grade tumors, highlighting their potential prognostic value [61,63]. However, GBM’s immunosuppressive microenvironment, marked by factors like PD-L1, IL-8, and TGF-β, hinders NK function [64,65]. A better understanding of the cytokine interplay between NK cells and GBM is essential for elucidating the prognosis. Strategies targeting factors like TGF-β show promise in overcoming the challenges posed by the tumor microenvironment [58,66]. Within the immunosuppressive tumor microenvironment of GBM, the prognostic role of NK cells is complex. While an immunosuppressive milieu typically characterizes GBM, studies highlight NK cells as potent effectors against brain tumors [58,65]. Notably, low numbers of NK cells infiltrate GBM, representing approximately 2% of the total immune cells [58,65,67]. However, their scarcity does not diminish their efficacy as evidenced by their strong impact on GBM and potential to prevent systemic metastasis [58,65,68]. Understanding NK cell-related gene signatures has the potential to offer insights into prognosis from a different therapeutic vantage point, paving the way for novel immunotherapeutic strategies in GBM [69].

### 2.4. Therapeutic Use of NK Cells

NK cells have broad implications and potential in the use of GBM treatment. Multiple immunotherapy modalities are designed to directly or indirectly solicit the antitumor activities of NK cells against glioblastoma. While this review is not comprehensive enough to cover the details of all therapies being explored, we seek to focus on the targets of several promising pathways. Some NK immunotherapies target NK interactions with GBM cells and others directly modify NK cells to target GBM. There are also varying ways in which to administer the therapies to patients: intravenously, intrathecally, intrathecally, or intracranially [61]. Since immune checkpoint inhibitors (ICIs) were introduced as a treatment for multiple types of cancer, there has been a scientific push to use ICIs in patients with glioblastoma. ICIs target molecular interactions (“immune checkpoints”) between GBM and immune cells (in this case, NK cells) which inhibit the antitumor functions of the immune cells. Most often, ICIs are monoclonal antibodies against the inhibitory ligand expressed on the cancer cell membrane or against the inhibitory receptor expressed on the NK cell surface [70,71]. Either way, when the inhibitory immune checkpoints are blocked, this pushes the NK cell towards activation in the sum of its activity. Examples of immune checkpoints that are relevant to NK cell activity include the PD-1/PD-L1 checkpoint, the TIGIT/CD155 immune checkpoint, the CD47 immune checkpoint, and the B7-H3 immune checkpoint. While advanced studies of immune checkpoint inhibitors have not yielded successful results so far, these studies should remain engaged [72]. Similar to ICIs, monoclonal antibodies targeted against antigens overexpressed on cancer cells are also being explored in the context of GBM [73]. These therapies are designed to bind to and block the activity of an advantageous GBM marker, yet the main function in interactions with NK cells is to stimulate antibody-dependent cellular cytotoxicity (ADCC). So far, none of these monoclonal antibodies have yielded significant survival benefits to patients due to various limitations [74]. Small interfering microRNAs (siRNAs) and some oncolytic viruses have been and are being studied for the potential to downregulate molecules on GBM cells which benefit GBM tumor cells in their interactions with NK cells [61]. siRNAs have been used to knock down certain molecular factors from being expressed to increase NK cytotoxic activity against the GBM cells [61,75]. Similarly, some researchers have used oncolytic viruses to target GBM cells for infection and subsequent changing of molecular phenotype to better stimulate NK anticancer functions [61,76]. Tumor-targeting vaccines have also been studied in GBM patients. While vaccines do not directly target NK cell activities, they target the activity of dendritic cells against the tumors, which in turn can influence NK cells towards being stimulated against the GBM tumor cells [61,77]. To date, no study has conclusively shown vaccines in GBM to benefit overall survival [78]. The other previously mentioned categories of NK cell immunotherapies specifically modify NK cell activity, not only the interactions with the environments of the NK cells. These phenotype modifications include ex vivo activation or pre-treatment via cytokines or genetic alterations. Ex vivo activation involves bathing NK cells in an extracellular milieu of cytokines (such as IL-2, IL-12, IL-15, IL-18, or IL-21) or other factors that activate the NK cells and lead to an increase in CD16 expression and antitumor cytokine production [79,80]. Genetic modification of NK cells involves changing the protein expression through a process of engineering [81]. Most commonly, this is exemplified by the use of chimeric antigen receptors (CARs). CARs are genetically constructed receptors designed to target a specific antigen leading to the activation of the CAR-expressing NK cell [82]. Currently, there are multiple studies evaluating the use of CAR-NK cells in malignant brain tumors [61,83,84]. Another alteration for purposes of therapy includes modifying the NK cell to be resistant to the immunosuppressive microenvironment through either a mechanism of constituent stimulatory activity or a mechanism of blocking inhibitory chemical signals such as TGF-β [14,66]. Whether cells are ex vivo activated or genetically engineered, they must be reintroduced into patients to be able to affect the tumor. There are several main routes of administration being explored, commonly either intravenous, intratumoral, intrathecal, or intracranial [9,61]. Each type of introduction of modified cells is being explored in preclinical studies and clinical trials. The source of the NK cells utilized may also vary. NK cells may be obtained via primary acquisition from healthy donors, from umbilical cord blood samples, and through expanded NK cell lines. The NK cell lines for therapeutic use are usually NK-92 and NK-92MI, with variations spinning off these two base cell lines. The NK-92 cell line is an immortalized cell line originating in a patient with lymphoma [85]. This cell line is active in performing NK cell functions and can be easily expanded but requires the addition of continuous IL-2. NK-92MI was developed to overcome the need for constant IL-2. NK-92MI is the NK-92 cell line with a transgene to produce its own IL-2 [86]. The NK-92MI cell line is immensely valuable for studying cancer immunotherapy in NK cells. NK-92MI cells can be grown for extended periods, are malleable for genetic changes such as the addition of a CAR molecule, and are useful due to their ability to perform in both in vitro and in vivo experimental settings. Both of these cell lines are very important in the development of NK cell-based immunotherapies for cancer, including GBM [85]. As many scientists have recognized before this moment, and regardless of the individual outcomes of each area of therapeutic study, it is likely the best way to move forward and utilize these treatments for patients will be in a combinatorial manner [61]. This will decrease the likelihood of tumors being able to escape destruction and potentially decrease the recurrence of tumors.

## 3. Current Targets for NK Cell-Based Immunotherapies for GBM

### 3.1. EGFR

**Figure 1:**
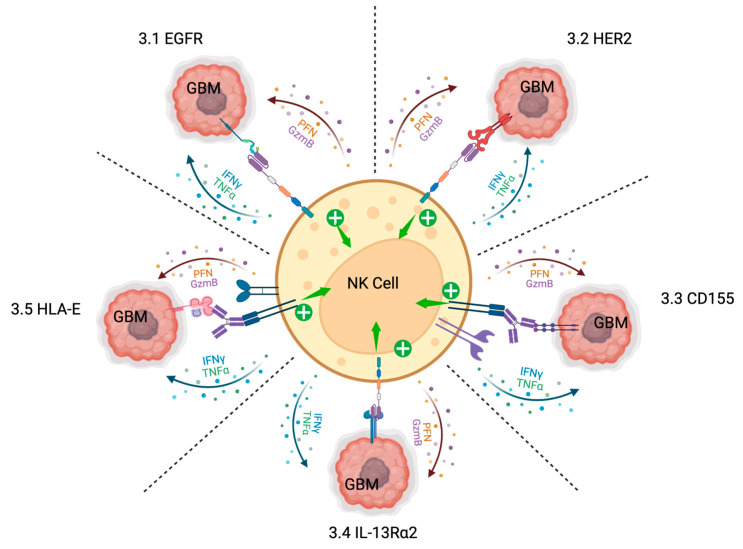
Diagram showing the referenced current NK cell targets and highlighted methods of stimulating NK activity. 3.1–EGFR: targeting EGFR expression on GBM cells with an anti-EGFR CAR stimulates NK cytotoxicity (“PFN” and “GzmB”) and cytokine production. 3.2–HER2: targeting GBM HER2 expression with an anti-HER2 CAR-NK cell. 3.3–CD155: blocking the CD155/TIGIT checkpoint with a monoclonal antibody and subsequently stimulating ADCC. 3.4–IL-13Rα2: targeting IL-13Rα2 with a CAR-NK cell to stimulate cytotoxicity and cytokine production. 3.5–HLA-E: blocking the HLA-E/NKG2A immune checkpoint with a monoclonal antibody and subsequent stimulation of ADCC for NK cytotoxicity and cytokine production. Created with BioRender.com.

Epidermal Growth Factor Receptor 1 (EGFR, ErbB1, or HER1) is a receptor tyrosine kinase that takes on a dimeric form upon binding one of its ligands and is one of the most common oncogenic mutations found in GBM [87,88,89]. EGFR activation and subsequent intracellular signaling leads to cell proliferation and survival, benefitting the GBM cancer cells [90]. Within the discussion of EGFR’s role in GBM, special consideration must be given to a very common genetic mutation: EGFR variant III (EGFRvIII) [91]. EGFRvIII contains a deletion which leads to the constitutive activity of the EGFR pathway, without the binding of ligand [88]. This mutation is very commonly seen in GBM and often occurs alongside EGFR amplification and overexpression in GBM [88,92]. Due to the big role EGFR plays in GBM proliferation and growth, and its very common overexpression and amplification in large amounts of patients, it is a frequent target for cancer therapies, especially for modern tyrosine kinase inhibitors [90,93]. Interestingly, it also plays a role in aiding GBM in promoting an immunosuppressive environment and in evading immune responses from T cells and NK cells [94,95]. Specifically, it promotes the expression of molecules that tilt the NK cell activity axis in favor of inhibition, which promotes GBM survival [90]. Due to this fact, it is important to make EGFR a target of NK cell immunotherapy for GBM. EGFR (including EGFRvIII) has been shown to influence the immunology of GBM in ways that favor the survival and proliferation of GBM tumors. To combat the immunosuppressive activity of EGFR, multiple modalities of immunotherapy have been attempted and more are being currently developed [65]. Immunotherapies against EGFR have been constructed with varying goals in mind: use EGFR as an activation target; inhibit EGFR signaling via blocking ligand binding; and use combination EGFR-targeting tyrosine kinase inhibitor drugs alongside other GBM immunotherapy strategies such as immune checkpoint inhibitors and CAR T cells [89,93,96,97,98]. To date, many of these avenues of therapy are still being explored. CAR-NK cells expressing CARs specific for EGFR and EGFRvIII have been studied in preclinical animal settings and are being considered for use in clinical studies (see Figure 1 below) [83,99].

### 3.2. HER2

Human Epidermal Growth Factor Receptor 2 (ErbB2, HER2, or Neu) is a receptor tyrosine kinase that is an oncogenic driver in many cancers, including GBM [100]. HER2 does not bind a ligand like EGFR does but can form dimers with other receptors (such as EGFR and ErbB3) and facilitate signaling [88,100]. Similar to EGFR, HER2 expression in GBM tumors supports tumor cell proliferation, survival, and angiogenesis [100,101]. HER2 is not typically expressed at high levels in the brains of healthy adults; it is only overexpressed in adults in the context of cancers [102]. HER2 overexpression and amplification in GBM are highly correlated with poor prognosis in patients with GBM [103,104]. Due to its large role in GBM tumorigenesis, HER2 is a frequently sought-after molecular target for designing immunotherapies. A recent study from a group at the University of Florida demonstrated the use of a novel mRNA vaccine to boost a robust immune response in patients with GBM [105]. The use of HER2-specific CARs for T cells and NK cells is also being explored. Multiple studies have been published investigating the use of HER2-targeted CAR-T cells and have shown success in in vitro and in vivo settings [106,107]. CAR-NK cells targeting HER2 in multiple cancers have been an exciting topic of discussion (see Figure 1 below). An immortalized cell line was recently developed which expresses an HER2-specific CAR on NK cells. This cell line, titled “NK-92/5.28.z”, contains a second-generation CAR molecule apparatus with a CD28 costimulatory domain and CD3ζ signaling domain [108]. Of great significance, this cell line is being studied in multiple cancer settings but is currently being tested in humans with glioblastoma by Burger et al. in a Phase I clinical trial and has so far been shown to be safe [109]. These current safety findings are in line with previous postulations that CAR-NK cells should be safer in patients due to no GVHD, no cytokine storm, and the off-the-shelf approach [109].

### 3.3. CD155

CD155 (also known as the Poliovirus Receptor or “PVR”) is an antigen that is overexpressed in the context of GBM [110]. CD155 has a diverse range of functions, the likes of which favor tumor growth and proliferation [111,112,113]. CD155 plays an important role in tumor cell interactions with NK cells mediated through several different NK receptors. Two of the receptors CD155 interacts with, CD226 and CD96, drive anti-tumor responses of NK cells [111,114]. However, CD155 on GBM cells also interacts with an inhibitory receptor on NK cells termed T cell immunoreceptor with Ig and ITIM domains (TIGIT) [115]. When bound by CD155, TIGIT inhibits NK cell antitumor activities and benefits the survival and proliferation of the tumor cells [111,115,116]. Because of the power of this inhibitory activity, the CD155-TIGIT immune checkpoint has been a focus of multiple studies investigating the possibility of multiple CD155/TIGIT-targeted immunotherapies against multiple cancers [84,111,117,118,119]. It has already been shown that blocking the inhibitory CD155/TIGIT checkpoint in the context of cancer leads to increased NK cell anticancer functioning against the tumor cells (see Figure 1) [120]. Similar to other targets, the targeting of the CD155/TIGIT immune checkpoint as a therapeutic basis for GBM is currently being evaluated in cell culture models and animal models [111]. As of the date of this publication, there are no clinical trials for targeting the CD155/TIGIT immune checkpoint for GBM.

### 3.4. IL-13Rα2

IL-13Rα2 is a subunit of the receptor for the interleukin IL-13. Normally, IL-13 binds with IL-13Rα1 on the surfaces of cells and mediates the intracellular signaling cascade that is important to the subsequent immune pathways [83,121]. However, GBM tumors have been shown to overexpress the IL-13Rα2 receptor subunit on their surfaces, which does not contain the same intracellular signaling cascade to induce antitumor immunity [122,123]. IL-13Rα2 does bind to IL-13 more readily than IL-13Rα1 and it is therefore hypothesized that GBM tumors potentially use IL-13Rα2 expression to bind up available IL-13 and prevent immune responses to the tumor cells by stimulating TGF-β production [83,121,124]. The fact that patients with higher expressions of IL-13Rα2 in GBM correlate with worse prognoses lends evidence to this hypothesis [83,125]. Given this and its general overexpression, researchers are investigating the potential of targeting IL-13Rα2 as the basis of immunotherapy using CAR-T cells, memory-enriched T cells, Bi-specific killer cell engagers (BiKEs), and functionalized nanocarriers [65,83,126,127]. Considering the attention already devoted to investigating T cell-based therapies targeting IL-13Rα2, many researchers suggest that the field should contemplate investigating the utility of targeting IL-13Rα2 with NK cell-based therapies, especially with CAR-NK cells (see Figure 1) [83]. To date, the only clinical studies evaluating the efficacy of targeting IL-13Rα2 are with the use of CAR-T cells [61]. The use of IL-13Rα2 for NK cell applications remains in the realm of preclinical studies in cell culture and animal models.

### 3.5. HLA-E

HLA-E is a nonclassical HLA class I molecule that has been shown in many studies to benefit cancer progression via immune escape [128]. When expressed on the surfaces of cancer cells, HLA-E has been shown to mediate inhibitory signals to NK cells via interactions with the NKG2A receptor [129,130]. The targeting of this NKG2A-HLA-E immune checkpoint in the development of cancer immunotherapies is also being discussed in the context of GBM (see Figure 1) [131]. Currently, there is a monoclonal antibody available (“monalizumab”) that targets NKG2A on NK cells and prevents the interaction with HLA-E, thus preventing the inhibitory signals to the NK cell [132]. HLA-E is expressed on GBM cells and to benefit the immune escape of GBM and GBM-stem cells [133,134]. The expression of HLA-E in gliomas has also been correlated with more aggressive pathological tumor grades in gliomas [135]. However, as with other NK targets, targeting the NKG2A-HLA-E immune checkpoint alone in GBM will likely not be enough to benefit patients. Studies evaluating the NKG2A/HLA-E immune checkpoint in GBM so far have been conducted in in vitro preclinical studies [131]. Preclinical in vivo studies and clinical patient studies evaluating this potential have not yet been conducted.

## 4. Prospective Targets for NK Cell-Based Immunotherapies for GBM

### 4.1. LLT1

**Figure 2:**
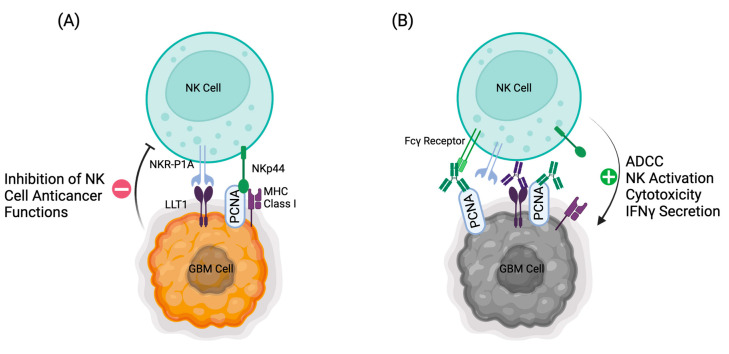
Schematic representation of the mechanism by which GBM escapes NK cell-mediated killing. (**A**) GBM expresses LLT1 and csPCNA and interacts with NKR-P1A (CD161) and NKp44, respectively, and delivers inhibitory signals to NK cells. (**B**) Proposed method of blocking the inhibitory signals using a monoclonal antibody as an immune checkpoint inhibitor for LLT1 and/or csPCNA. Created with BioRender.com.

Lectin-like Transcript 1 (LLT1, OCIL, or CLEC2D) is a protein typically found in humans on immune cells, where it often forms a homodimer between NK cells and other immune cells [136]. While cloning of LLT1 initially led researchers to believe it did not contain inherent inhibitory capacity, LLT1 has also been shown to act as a ligand for the NK cell receptor CD161 (NKR-P1A) where it can lead to an inhibitory response in the NK cell being acted upon [137,138,139]. Thus, this interaction has been termed the “LLT1-CD161 immune checkpoint” [140]. Several in vitro studies have demonstrated that blocking this immune checkpoint from occurring in interactions between cancer cells and NK cells can lead to increased NK cell anticancer activity against the target cancer cells. This interaction has been studied and demonstrated in the context of triple-negative breast cancer, prostate cancer, colorectal cancer, and glioma [141,142,143,144]. LLT1 expression has been demonstrated in the contexts of oral squamous cell cancer, non-small cell lung cancer (NSCLC), urothelial cancers, and multiple leukemias and lymphomas [140,145,146,147]. The utility of blocking the CD161-LLT1 immune checkpoint in T cells has also been demonstrated [147]. The prospect of blocking this immune checkpoint from inhibiting NK cell anticancer functions and in turn activating the NK cells against the cancer makes it an attractive target, given that it has also been previously identified in the context of gliomas (see Figure 2). Roth et al. have demonstrated that multiple glioma cell lines and patient tissue samples are positive for the expression of LLT1 [144]. Thus, it is reasonable to make LLT1 a focus of study for the potential of anti-glioblastoma NK cell immunotherapy modalities.

### 4.2. Cell Surface PCNA (csPCNA)

Proliferating cell nuclear antigen (PCNA) was first identified as the sliding clamp for DNA polymerase in the nucleus in a homotrimer [148,149,150,151]. It is a highly conserved protein that plays a hugely important role in DNA replication and cell cycle regulation when confined to the nucleus [150,151,152]. It also plays an important role in DNA repair in the nucleus [153]. It is also highly expressed in many cancers due to its important role in rapidly dividing cells [154]. However, it has also been demonstrated that PCNA can be expressed on the surfaces of cells as a monomer where it forms a complex with HLA class I molecules and mediates inhibitory interactions to NK cells via the NKp44 receptor, forming another immune checkpoint [155,156,157]. From here on, we will distinguish PCNA expressed on the cell surface as “csPCNA.” The NKp44-csPCNA immune checkpoint has been demonstrated to play an important role in inhibiting NK cell anticancer activity. On the flip side of this, blocking the NKp44-csPCNA immune checkpoint has been demonstrated to be a potentially effective immunotherapeutic strategy in the contexts of multiple kinds of cancer, including breast cancer, lymphoma, leukemia, prostate cancer, cervical cancer, colorectal cancer, multiple myeloma, and, of significance, glioblastoma [143,155,157,158,159,160]. Due to the significant role csPCNA plays in allowing cancers to survive, and that one well-characterized human glioblastoma cell line has been shown to express csPCNA in interactions with NK cells, it is reasonable to suggest that some glioblastoma tumors might express csPCNA as a means of evading immune functions, especially in interactions with NK cells [155]. Given these facts, we propose csPCNA as a target of further study for immune checkpoint inhibition using monoclonal antibody therapy and other modalities of NK cell immunotherapy, such as BiKEs and CAR-NK cells (see Figure 2).

## 5. Conclusions

GBM is the most common type of malignant brain tumor in adults and has a dreadful overall survival. There has been a strong push from researchers to be creative in implementing the immunotherapy revolution into GBM treatment, so far with minimal success. NK cell-based immunotherapies are a promising approach to treating GBM from multiple strategic angles: monoclonal antibody therapy targeting TAAs, immune checkpoints, and pure ADCC; CAR-NK cell therapy and other adoptive NK therapies; and Bi-specific killer cell engagers (BiKEs). NK therapies often require focusing on specific tumor antigens for targeting. Here, we reviewed some of the common targets being looked at in GBM immunotherapy relevant to NK cells: EGFR, HER2, CD155, IL-13Rα2, and HLA-E. Following this, the main objective of our paper is to propose the possibility of investigating the use of LLT1 and csPCNA as options for NK cell-based immunotherapy.
